# Psychotherapy for adult depression in low- and middle-income countries: an updated systematic review and meta-analysis

**DOI:** 10.1017/S0033291723002246

**Published:** 2023-12

**Authors:** Lingyao Tong, Clara Miguel, Olga-Maria Panagiotopoulou, Eirini Karyotaki, Pim Cuijpers

**Affiliations:** 1 Department of Clinical, Neuro & Developmental Psychology, Amsterdam Public Health Research Institute, Vrije Universiteit Amsterdam, Amsterdam, the Netherlands; 2 WHO Collaborating Centre for Research and Dissemination of Psychological Interventions, Vrije Universiteit Amsterdam, Amsterdam, the Netherlands; 3 Babeș-Bolyai University, International Institute for Psychotherapy, Cluj-Napoca, Romania

**Keywords:** Depression, low- and middle-income countries, meta-analysis, non-Western countries, psychotherapy

## Abstract

Previous meta-analyses on psychotherapy for adult depression have found a larger treatment effect in non-Western trials compared to Western trials (i.e. North America, Europe, and Australia). However, factors contributing to this difference remain unclear. This study investigated different study characteristics between Western and non-Western trials and examined their association with effect size estimates. We systematically searched PubMed, PsycINFO, Embase, and Cochrane Library (01–09–2022). We included randomized-controlled trials (RCTs) that compared psychotherapy with a control condition. The validity of included RCTs was assessed by the Cochrane risk of bias assessment tool (RoB 1). Effect sizes were pooled using the random-effects model. Subgroup analyses and meta-regressions were also conducted. We identified 405 eligible trials, among which 105 trials (117 comparisons, 16 304 participants) were from non-Western countries. We confirmed that non-Western trials had a larger treatment effect (*g* = 1.10, 95% CI 0.90–1.31) than Western trials (*g* = 0.57, 95% CI 0.52–0.62). Trials from non-Western countries also had more usual care controls, higher risk of bias, larger sample sizes, lower mean ages, younger adults, more group-based interventions, and other recruitment methods (e.g. systematic screening; *p* < 0.05). The larger effect sizes found in non-Western trials were related to the presence of wait-list controls, high risk of bias, cognitive-behavioral therapy, and clinician-diagnosed depression (*p* < 0.05). The larger treatment effects observed in non-Western trials may result from the high heterogeneous study design and relatively low validity. Further research on long-term effects, adolescent groups, and individual-level data are still needed.

Psychological interventions have demonstrated efficacy in treating adult depression, and the estimation of treatment effect sizes can be influenced by various study characteristics, including study design and validity (Cuijpers, 2017; Munder et al., 2019). Meta-analyses of randomized controlled trials (RCTs) focusing on adult depression have found smaller effect sizes among individuals with chronic depression or comorbid substance abuse (Cuijpers, 2017). However, when examining specific populations such as college students, older adults, or individuals with general medical diseases, the effect sizes were comparable (Cuijpers, 2017; Cuijpers et al., 2016). The validity of trials can also significantly impact effect size estimates, as studies with a high risk of bias (indicating low validity) tend to overestimate the treatment outcome, leading to larger effect sizes (Cuijpers, Karyotaki, Reijnders, Purgato, & Barbui, 2018).

In a recent systematic review and meta-analysis focusing on psychotherapy for adult depression, it was found that trials conducted in non-Western countries yielded a larger effect size compared to those in Western countries (i.e. North America, Europe, or Australia; Cuijpers et al., 2018). However, it is essential to interpret the findings of this meta-analysis with caution, as the number of trials conducted in non-Western countries and subgroup analyses was relatively small (32 studies). Thus, the observed larger effect size in non-Western trials may indeed reflect a genuine difference, suggesting a potentially more effective intervention; however, it can also result from the different study characteristics between Western and non-Western trials (Cuijpers, 2017; Cuijpers, Li, Hofmann, & Andersson, 2010). To date, no study has comprehensively examined the variations in study characteristics between Western and non-Western trials and their potential associations with effect estimates.

As the number of RCTs investigating psychotherapy for adult depression continues to increase exponentially, we conducted an updated systematic review and meta-analysis of the previously published paper (Cuijpers et al., 2018). By including double the number of trials from non-Western countries, the current study benefits from improved statistical power. It is also the first study that explored differences in study characteristics and identified factors contributing to the larger effect sizes observed in non-Western trials. Overall, the objectives of this study were threefold: (1) to update the findings from the previous meta-analysis and compare treatment effects between Western and non-Western trials, (2) to explore variations in study characteristics between Western and non-Western trials, and (3) to examine how these differences might account for the larger effect size observed in non-Western trials.

## Methods

### Identification and selection of studies

Potential papers were included using an existing database of RCTs on psychotherapy for depression (https://osf.io/cdfu2). This database was developed through systematic searching in four databases (PubMed, PsycINFO, Embase, and Cochrane Central Register of Controlled Trials) and from other sources, such as searching in reference lists of previous meta-analyses, contacting other researchers, and identifying studies from other databases. The latest search was conducted until September 2022. Details of the databases and searching methods were previously described (Cuijpers, Straten, Andersson, & Oppen, 2009). The most up-to-date details and publicly available data used in this study can be found on the project website (www.metapsy.org). The full search strings for PubMed are provided in the online Appendix.

All records were screened by two independent researchers based on a pre-agreed inclusion criterion. Titles and abstracts were double-blind screened in Rayyan. If one of the researchers thought the study was qualified, the full text was retrieved to Endnote to decide whether the study was eligible. Any discrepancies between the two researchers during the screening and inclusion process were solved through discussion.

#### Inclusion and exclusion criterion

The general eligibility criteria for the existing meta-analytic database were described previously (https://osf.io/cdfu2). For the current study, we included RCTs on adult depression that compared psychotherapy to an inactive control group, which encompassed wait-list (WL), care-as-usual (CAU), and other inactive controls such as psychoeducation and counseling. The diagnoses of depression were determined through valid questionnaires or clinical interviews. Any type of psychological intervention delivered in any format was eligible for inclusion, except for the completely self-guided format. Previous research has demonstrated that the effects found in self-guided trials were significantly lower than other formats (Cuijpers, Noma, Karyotaki, Cipriani, & Furukawa, 2019).

We excluded studies that recruited participants from inpatient settings because previous findings indicated smaller effects in this specific group (Cuijpers et al., 2018, 2021a). We also excluded maintenance studies in which participants had partly or fully recovered from depression through early treatment. Papers published before 2000 were excluded from this study, as most of the studies conducted in non-Western countries (excluding North America, Europe, and Australia) were published after 2000.

### Quality assessment and data extraction

We used the Cochrane risk of bias assessment tool (RoB 1) to evaluate the quality of the included RCTs (Higgins et al., 2011). Four potential sources of risk of bias were assessed, including randomization sequence generation, allocation concealment, assessment blindness, and handling of missing data. The missing data were considered positive when analyzed using the intention-to-treatment (ITT) approach, which means that all randomized patients were included in the analyses (Gupta, 2011; Higgins et al., 2011). A low risk of bias indicated that all four potential sources of risk of bias were deemed positive, while a high risk of bias indicated that only one or none of the potential sources were positive. The assessment was conducted independently by two researchers, and any disagreements were solved through discussion.

Data regarding study characteristics were extracted and classified into three categories: characteristics of trials (type of control group, region of countries, income level of countries, and risk of bias), participants (sample size, age category, mean age, the proportion of women, target group, diagnosis method, and recruitment method), and treatment (type, format, and the number of sessions). Two independent researchers extracted the data, and data accuracy and consistency were checked between them.

The income level of countries was categorized into low/lower-middle-income countries (LMICs), upper-middle-income countries, and high-income countries (HICs) according to the World Bank (http://data.worldbank.org), taking into account the year of publication. The region of countries was categorized based on the seven regions from the World Bank: North America, East Asia and Pacific, Europe and Central Asia, Latin America and the Caribbean, the Middle East and North Africa, South Asia, and Sub-Saharan Africa. Additionally, we classified the regions geographically as North America, Europe, Oceania, Asia, Africa, and Latin America (including the Caribbean).

For studies conducted in non-Western countries, we also examined whether the intervention was culturally adapted to the local settings and the population. We considered the intervention was adapted if (1) the authors explicitly stated that the study used adaptions or (2) the intended interventions were developed based on local conditions, models, or theories in non-Western countries (Cuijpers et al., 2018). If there were no implications of adaptions or only indications of language translations, we considered the intervention not culturally adapted.

### Primary outcome

The primary outcome was the difference in depression between the intervention and control groups at post-assessment. These effects were expressed using Hedges' *g*, as some RCTs had a relatively small sample size (Enzmann, 2015). A *g* score below 0.2 indicates small effects, 0.5 is moderate, and 0.8 is large (Cohen, 1988). We extracted the means (*M*) and standard deviations (s.d.) of each intervention and control group at post-assessment where the diagnoses or symptoms of depression were assessed. We also extracted dichotomous variables (i.e. remission rate, response rate) or other statistics (i.e. *t* value or *p* value) when the mean differences were not reported.

### Data analyses

The differences in study characteristics between Western and non-Western countries were performed in IBM SPSS (version 26). Independent *t* tests were used to examine the differences for continuous variables, and χ^2^ tests were used for categorical variables.

The effect sizes were pooled in R studio (Version 2022.07.1 for macOS) using the metapsyTools package (Version 1.0.10; Harrer, Kuper, Sprenger, & Cuijpers, 2022). The full R scripts were provided in the online Appendix. This package imports the functionality of the meta, metafor, and dmetar package (Balduzzi, Rücker, & Schwarzer, 2019; Harrer, Cuijpers, Furukawa, & Ebert, 2019; Viechtbauer, 2010), and it was specifically developed for the meta-analytic project of which this study is part. We pooled effect sizes using different methods implemented in metapsyTools and examined whether different pooling methods resulted in different outcomes. We selected the combined model as the main result, meaning that multiple effect sizes within one comparison were first pooled in one study and then pooled across studies and comparisons. An intra-study correlation coefficient of *ρ* = 0.5 was assumed when aggregating the within-comparisons effects. Besides, we conducted several alternative analyses to examine whether the main results were robust. First, we estimated the pooled effect only using the lowest or the highest effect sizes within one study. Second, we conducted the analyses excluding potential outliers (studies whose 95% CI did not overlap with the 95% CI of the pooled effect sizes) or extreme outliers (studies with a *g* > 2). Third, we pooled effect sizes for studies with a relatively low risk of bias (rob > 2). Last, we did the analyses using the multiple-level (three-level) correlated and hierarchical effects (CHE) model, which assumed that effect sizes were nested in studies and effects within studies are correlated (correlation coefficient *ρ* = 0.6; Pustejovsky & Tipton, 2022).

The random-effects model was used because of the assumed high heterogeneity among the included studies. The degree of heterogeneity in the effect sizes was assessed using *I^2^* with its 95% CI, where an *I^2^* score of 25% indicated low heterogeneity, 50% indicated moderate, and 75% indicated thigh heterogeneity (Higgins, Thompson, Deeks, & Altman, 2003). Additionally, we calculated the prediction interval (PI) to estimate the interval within which further observations are likely to fall (IntHout, Ioannidis, Rovers, & Goeman, 2016). The numbers-needed-to-be-treated (NNT) were calculated using the Furukawa formulae, with the control group's event rate conservatively set as 19% (based on the pooled response rate of 50% reduction of symptoms across trials in psychotherapies for depression) (Cuijpers et al., 2014; Furukawa, 1999). The NNT represents the number of patients needed to be treated to prevent an additional negative outcome. Publication bias was assessed by examining the funnel plot and employing various statistical methods, including Duval and Tweedie's trim and fill procedure, Rücker's limit meta-analyses, and the three-parameter selection model (Duval & Tweedie, 2000; McShane, Böckenholt, & Hansen, 2016; Rücker, Schwarzer, Carpenter, Binder, & Schumacher, 2011). Egger's tests were also conducted to assess the significance of bias captured by the funnel plot (Egger, Smith, Schneider, & Minder, 1997).

We conducted a series of subgroup analyses for categorical variables. The mixed-effects model was employed, where effect sizes within subgroups were pooled using a random-effects model, while differences between subgroups were tested using a fixed-effects model. Moreover, we used univariate meta-regression analyses to test the association between continuous variables (e.g. risk of bias and the year of publication) and effect sizes (dependent variable). Multivariate meta-regression analyses were used to test the effect of multiple study characteristics on treatment effect sizes. Three models were created, with treatment effect size as the dependent variable. In model 1, we included a dummy variable indicating whether the studies were conducted in Western or non-Western countries and included multiple study characteristics as predictors. In model 2, we used the same predictors, except that we replaced the dummy variable (Western *vs.* non-Western) with a variable indicating the region of countries classified by the World Bank. Similarly, model 3 examined the income level of countries. To avoid over-fitting, we further conducted parsimonious multivariate meta-regression analyses, in which we manually removed the least significant predictor step-wise until only the significant predictors remained in each of the three models.

## Results

### Selection and inclusion of studies

**Fig. 1.**
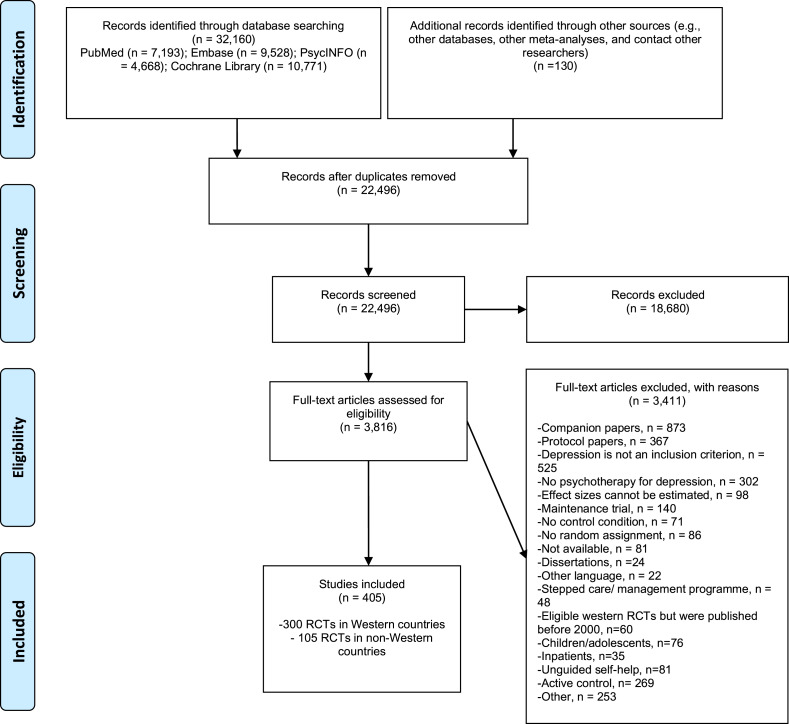
PRISMA flow diagram.

We identified 32 162 records (22 496 after duplicates were removed) and retrieved 3816 full texts for further consideration. We excluded 3411 studies because they did not meet the inclusion criterion for the current study. The PRISMA flowchart of the selection process and exclusion reasons are presented in Fig. 1. A total of 405 RCTs were included, with 105 studies (117 comparisons between an intervention and control group) from non-Western countries and 300 studies (329 comparisons) from Western countries.

### Characteristics of RCTs in non-Western countries

The selected characteristics of included studies from non-Western countries can be found in the online Appendix (Supplementary Table S1). In the 105 non-Western trials, the total number of participants was 16 304 (7995 in the intervention group and 8309 in the control group). Eighty-three studies were conducted in Asia (79.0%), fifteen (14.3%) were in Africa, and seven (6.7%) were in Latin America and the Caribbean. There were 25 studies (23.8%) from HICs, 40 studies (38.1%) from upper-middle-income countries, and 40 studies from LMICs. The risk of bias assessments showed that 22 comparisons (18.8%) were at high risk of bias, and 36 comparisons (30.8%) were at low risk of bias.

Participants were mainly recruited by other methods, such as systematic screening or recruitment from known patients in general medical settings (63 studies, 60.0%). They were also recruited from clinical settings (28 studies, 26.7%) and communities (14 studies, 13.3%). More than half of the trials (61 comparisons, 52.1%) used CBT as an intervention, 15 comparisons (12.0%) were behavioral activation therapy, 12 (10.3%) were third-wave therapy, and the remaining (*N* = 18, 23.0%) were other types of psychotherapy (i.e. psychodynamic therapy, interpersonal therapy, positive psychology therapy, life review therapy, supportive therapy, and other types of psychotherapy that did not correspond with the major types described above). Most psychotherapies were delivered in group formats (64 comparisons, 54.7%). Thirty-two comparisons (27.4%) were delivered in individual formats, 12 (10.3%) were in guided self-help formats, and 9 (7.8%) were in other formats (i.e. telephone-based, couple therapy, or mixed). A visual overview of included trials showing various study characteristics across countries can be seen in the online Appendix as an interactive map.

### Comparisons of study characteristics in Western *vs.* non-Western countries

Table 1 presents the significance test on differences in study characteristics between Western and non-Western trials. As we can see, compared with studies from Western countries, non-Western trials more often used CAU as a control group (χ^2^ = 7.19, *p* < 0.05). The risk of bias in non-Western trials was high (χ^2^ = 7.29, *p* < 0.05), owing to the inappropriate analyses with missing data (less ITT was used, χ^2^ = 25.90, *p* < 0.001). Non-Western trials also had a larger sample size (*t* = −3.08, df = 397, *p* < 0.05), a lower mean age (*t* = 4.69, df = 396, *p* < 0.001), and younger adult groups (mean age 18 to 24, χ^2^ = 19.04, *p* < 0.001). Participants in non-Western trials were mostly recruited from other settings and hardly from the communities (χ^2^ = 40.75, *p* < 0.001). Interventions delivered in non-Western trials were more often group based rather than individual based, the latter being mainly used in Western trials (χ^2^ = 45.75, *p* < 0.001).

**Table 1.** Significance test of difference in study characteristics between Western and non-Western trials

Studies’ characteristics			Non-western countries *N* (%)/*M* (s.d.)	Western countries *N* (%)/*M* (s.d.)	Tests
*N* studies			105 (25.9%)	300 (74.1)	
*N* comparisons			117 (23.8%)	353 (76.2%)	
Trials	Control group	CAU	71 (60.7%)	167 (48.6%)	χ^2^ = 7.19 ***p*** **=** **0.027**
		WL	28 (23.9%)	128 (37.1%)	
		Other	18 (15.4%)	58 (14.3%)	
	Risk of bias	High (0–1)	22 (18.8%)	40 (11.3%)	χ^2^ = 7.29 ***p*** **=** **0.026**
		Unclear (2–3)	59 (50.4%)	162 (45.9%)	
		Low (4)	36 (30.8%)	151 (42.8%)	
Participants	*N* Total		*M* = 142.00 (s.d. = 183.75)	*M* = 99.95 (s.d. = 87.17)	*t* (397) = −3.08 ***p*** **=** **0.026**
	Age category	Younger adults	13 (12.4%)	8 (2.7%)	χ^2^ = 19.04 ***p*** **<** **0.001**
		Adults	78 (74.3%)	219 (73.0%)	
		Older adults	13 (12.4%)	71 (23.7%)	
	Mean age		*M* = 38.41 (s.d. = 14.19)	*M* = 46.40 (s.d. = 14.03)	*t* (378) = 4.69 ***p*** **<** **0.001**
	Proportion of women		*M* = 73.44% (s.d. = 0.23)	*M* = 71.96% (s.d. = 0.22)	*t* (396) = −0.57 *p* = 0.570
	Target group	Unselected adults	32 (30.5%)	111 (37.0%)	χ^2^ = 2.14 *p* = 0.543
		Women with PPD	18 (17.1%)	39 (13.0%)	
		General medical	25 (23.8%)	73 (24.3%)	
		Other^a^	30 (28.6%)	77 (25.7%)	
	Diagnoses	Cut-off	52 (49.5%)	141 (47.0%)	χ^2^ = 1.98 *p* = 0.371
		Depressive disorder^b^	44 (41.9%)	143 (47.7%)	
		Sub-clinical	9 (8.6%)	16 (5.3%)	
	Recruitment	Clinical	28 (26.7%)	66 (22.0%)	χ^2^ = 40.75 ***p*** **<** **0.001**
		Community	14 (13.3%)	141 (47.0%)	
		Other	63 (60.0%)	93 (31.0%)	
Treatment	Type	CBT	61 (52.1%)	175 (49.6%)	χ^2^ = 0.23 *p* = 0.631
		Other	56 (47.9%)	178 (50.4%)	
	Format	Group	64 (54.7%)	79 (22.4%)	χ^2^ = 45.75 ***p*** **<** **0.001**
		Individual	32 (27.4%)	132 (37.4%)	
		Guided self-help	12 (10.3%)	95 (26.9%)	
		Other^c^	9 (7.7%)	47 (13.3%)	
	*N* Sessions		*M* = 8.87 (s.d. = 3.55)	*M* = 8.80 (s.d. = 5.97)	*t* (403) = −0.14 *p* = 0.886

CAU, care as usual; WL, wait-list; PPD, postpartum depression; CBT, cognitive behavioral therapy.

a Other = older adults, students or other population that do not correspond with the major category.

b Depressive disorders = clinician-rated major depression disorders, mood disorders, and chronic depression.

c Other = telephone-based therapy, couple therapy, or mixed format of therapy.

None of the remaining variables were significantly different between Western and non-Western trials, including the proportion of women, target group, diagnosis method, type of treatment, and the number of treatment sessions. We conducted further analyses comparing study characteristics between HICs and LMICs, revealing that the significance disappeared only for the control group and the risk of bias (see online Supplementary Table S2 in the Appendix).

### Effects of psychotherapy in non-Western countries

As can be seen from Table 2, we found a very large effect size (*g* = 1.10, 95% CI 0.90–1.31) with an NNT of 2.48 in non-Western trials. The heterogeneity of these studies was very high (*I*^2^ = 91.07%, 95% CI 89.80–92.17; PI: −0.80 to 3.00), showing a high variation of the effect sizes between studies. Forty-one studies (43 comparisons) did not overlap with the pooled effect sizes in their 95% CI and were considered potential outliers (for the forest plot, see the online Appendix). After excluding the outliers, the effect sizes dropped to *g* = 0.95 (95% CI 0.86–1.04; *I*^2^ = 53.45%, 95% CI 39.37–64.27; NNT = 2.91). Twelve studies (13 comparisons) had extremely high effect sizes (*g* > 2) and were considered extreme outliers. The effect size dropped to *g* = 0.79 (95% CI 0.70–0.89; *I*^2^ = 84.78%, 95% CI 82.07–87.09; NNT = 3.56) after excluding these studies. Through visual inspection of the funnel plot, we found considerable publication bias, with Egger's test also pointed a significant asymmetry (intercept: 2.73; 95% CI 1.61–3.85; *p* < 0.001). After correcting for the publication bias, such as using the Trim and fill method, the effect sizes dropped to *g* = 0.60 (95% CI 0.34–0.87; *n* = 36 studies added, Table 2).

**Table 2.** Overall effects on psychotherapy for adult depression compared with control conditions at post-assessment in non-Western countries

	*N*	*g*	95% CI	*p*	*I*^2^	95% CI	PI	NNT
Combined	117	1.10	0.90–1.31	<0.001	91.07	89.80–92.17	−0.80 to 3.00	2.48
One ES/study (lowest only)	105	1.03	0.81–1.26	<0.001	90.52	89.07–91.78	−0.95 to 3.02	2.66
One ES/study (highest only)	105	1.24	0.94–1.54	<0.001	92.55	91.49–93.47	−1.28 to 3.76	2.20
Outliers removed	74	0.95	0.86–1.04	<0.001	53.45	39.37–64.27	0.45–1.45	2.91
Extreme outliers (*g* > 2) removed	104	0.79	0.70–0.89	<0.001	84.78	82.07–87.09	−0.06 to 1.65	3.56
Only rob > 2	94	1.05	0.86–1.24	<0.001	92.94	91.90–93.85	−0.68 to 2.78	2.61
Three-level model (CHE)	153	1.16	0.93–1.40	<0.001	97.60	–	−1.28 to 3.61	2.35
Publication bias correction								
Trim-and-fill method (36 studies added)	153	0.60	0.34–0.87	<0.001	94.07	93.44–94.65	−2.34 to 3.55	4.86
Limit meta-analysis	117	0.42	0.18–0.65	<0.001	70.93		−1.49 to 2.32	7.4
Selection model	117	0.49			98.39	96.80–99.20		6.1

ES, effect size; *N*, number of comparisons; CI, confidence interval; PI, prediction interval; NNT, numbers-needed-to-be-treated; rob, risk of bias.

#### Subgroup analysis

Considering the extreme outliers found in non-Western trials, we conducted sensitive analyses comparing the results with and without the inclusion of extreme outliers. In the results that included extreme outliers (Table 3), we found significant differences in geographic regions, World Bank regions, risk of bias, and treatment format. Specifically, studies from Sub-Saharan Africa had the highest effect size (*g* = 1.82, 95% CI 0.39–3.25) than other regions (*g* = 0.64 to 1.51, *p* < 0.01). Low-risk-of-bias studies had the lowest effects sizes (*g* = 0.69, 95% CI 0.49–0.89) comparing to high-risk-of-bias studies (*g* = 1.93, 95% CI 1.13–2.72) and unclear-risk-of-bias studies (*g* = 1.06, 95% CI 0.82–1.30). Group-based psychotherapies (*g* = 1.28, 95% CI 0.99–1.58) showed larger effect sizes than individual-based psychotherapies (*g* = 1.02, 95% CI 0.54–1.51), guided psychotherapies (*g* = 0.71, 95% CI 0.41–1.01) or other formats of psychotherapies (e.g. telephone-based; *g* = 0.80, 95% CI 0.37–1.23, *p* < 0.05).

**Table 3.** Subgroup analyses of psychotherapy for adult depression compared with control conditions at pos-assessment in non-Western countries

		*N*	*g*	95% CI	*I*^2^	95% CI	NNT	*p*
Geographic region	Asia	92	1.08	0.89–1.26	90.3	88.8–91.7	2.54	**0.014**
	Latin America	9	0.64	0.31–0.96	77.0	56.1–87.9	4.54	
	Africa	16	1.82	0.39–3.25	94.5	92.5–96.0	1.57	
World Bank region	East Asia and Pacific	50	0.92	0.67–1.16	90.4	88.2–92.2	3.02	**0.004**
	Europe and Central Asia	4	1.14	−0.06 to 2.34	84.3	60.6–93.7	2.40	
	Middle East and North Africa	26	1.51	1.14–1.87	86.8	81.8–90.4	1.82	
	South Asia	12	0.85	0.45–1.25	94.5	92.1–96.2	3.30	
	Latin America and Caribbean	9	0.64	0.31–0.96	77.0	56.1–87.9	4.54	
	Sub-Saharan Africa	16	1.82	0.39–3.25	94.5	92.5–96.0	1.57	
Income level of country	High	27	0.81	0.50–1.11	84.9	79.1–89.1	3.48	0.092
	Upper middle	47	1.02	0.79–1.25	90.2	87.9–92.1	2.70	
	Low/lower middle	43	1.45	0.93–1.97	93.3	91.8–94.5	1.89	
Risk of bias	High (0–1)	22	1.93	1.13–2.72	94.5	92.8–95.8	1.51	**0.001**
	Unclear (2–3)	59	1.06	0.82–1.30	85.8	82.4–88.5	2.59	
	Low (4)	36	0.69	0.49–0.89	89.0	85.8–91.5	4.17	
Control group	CAU	71	0.94	0.74–1.13	90.4	88.5–91.9	2.95	0.136
	WL	28	1.38	0.93–1.82	90.2	87.1–92.6	1.98	
	Other	18	1.33	0.42–2.24	93.0	90.4–94.9	2.05	
Age group	Adults	88	1.13	0.85–1.40	92.5	91.3–93.5	2.42	0.954
	Younger adults	15	1.06	0.70–1.42	80.1	68.0–87.6	2.59	
	Older adults	13	1.12	0.77–1.47	77.3	61.5–86.6	2.44	
Recruitment	Clinical	33	0.98	0.62–1.35	90.9	88.3–93.0	2.82	0.533
	Community	17	0.99	0.68–1.31	87.7	81.9–91.7	2.79	
	Other	67	1.21	0.89–1.53	91.6	90.0–92.9	2.25	
Target group	Unselected adults	38	0.96	0.65–1.26	90.8	88.3–92.7	2.88	0.086
	Women with PPD	18	0.75	0.44–1.05	94.6	92.8–96.0	3.80	
	General medical	28	1.53	0.92–2.13	93.1	91.1–94.6	1.80	
	Other	33	1.13	0.75–1.50	83.4	77.6–87.7	2.42	
Diagnosis	Cut-off	57	1.18	0.80–1.57	90.7	88.7–92.3	2.31	0.484
	Depressive disorder	49	1.11	0.85–1.36	89.8	87.4–91.8	2.46	
	Subclinical	11	0.84	0.34–1.35	94.0	91.1–96.0	3.34	
Cultural adaption	Yes	44	0.93	0.66–1.20	93.1	91.6–94.4	2.98	0.143
	No	73	1.22	0.93–1.52	89.0	86.9–90.8	2.24	
Type of therapy	CBT	61	1.30	0.93–1.67	92.6	91.3–93.8	2.10	0.060
	Other	56	0.90	0.70–1.11	87.2	84.1–89.6	3.09	
Format of therapy	Group	64	1.28	0.99–1.58	91.3	89.6–92.7	2.13	**0.030**
	Individual	32	1.02	0.54–1.51	89.0	85.6–91.6	2.70	
	Guided self-help	12	0.71	0.41–1.01	90.9	86.0–94.1	4.04	
	Other	9	0.80	0.37–1.23	90.9	85.1–94.5	3.53	

*N*, numbers of comparisons; CI, confidence interval; NNT, numbers-needed-to-be-treated; WL, wait-list; CAU, care as usual; CBT, cognitive behavior therapy; PPD, postpartum depression.

However, the results were different in the analyses without extreme outliers (see online Supplementary Table S3, Appendix). Significant differences were additionally observed in the control group, age group, and cultural adaption (*p* < 0.05); however, they disappeared for geographic region and treatment format. Specifically, studies that compared interventions to WL (*g =* 0.93, 95% CI 0.73–1.12) or CAU *(g =* 0.81, 95% CI 0.68–0.94) had larger effect sizes than other controls (*g =* 0.55 95% CI 0.37–0.73, *p* < 0.01). Younger adults (mean age = 18 to 24, *g* = 0.91, 95% CI 0.63–1.19) and older adults (mean age > 55, *g =* 1.01, 95% CI 0.70–1.31) had larger effect sizes than other adults (*g =* 0.75, 95% CI 0.63–0.86, *p* < 0.05). Interventions with cultural adaption showed lower effects (*g =* 0.67, 95% CI 0.54–0.79) than no adaptions (*g =* 0.90, 95% CI 0.76–1.03, *p* < 0.05).

Lastly, univariate meta-regression analyses showed significant negative associations between risk of bias and treatment effects (coefficient: −0.34; *p* < 0.01). No other significant associations were found for mean age, percentage of women, number of treatment sessions, or the year of publication.

### Effects of psychotherapy in Western *vs.* non-Western countries

In Table 4, we found a moderate treatment effect size in Western trials (*g* = 0.57; 95% CI 0.52–0.62; NNT = 5.18), which is significantly smaller than in non-Western trials (*g* = 1.10, *p* < 0.001). The heterogeneity of included Western trials was high (*I*^2^ = 75.0; 95% CI 72.30–77.40), but it was smaller than non-western trials (*I*^2^ = 91.1). Ten (14 comparison) studies had extremely high effect sizes (*g* > 2). Studies from Europe and Central Asia (*g* = 0.55; 95% CI 0.48–0.63) and North America (*g* = 0.57; 95% CI 0.50–0.65) had the smallest effect sizes, whereas Sub-Sahara Africa (*g* = 1.82; 95% CI 0.39–3.25) and the Middle East and North Africa (*g* = 1.51; 95% CI 1.14–1.87) had the highest effect sizes. Similar to the subgroup analysis findings in non-Western trials, high-income trials had the lowest effect size (*g* = 0.59; 95% CI 0.53–0.64) compared with upper-middle-income trials (*g* = 1.00; 95% CI 0.78–1.21) and low/lower-income trials (*g* = 1.45; 95% CI 0.93–1.97). Lastly, we conducted a separate subgroup analysis in which the HICs were further classified into high-income Western countries and high-income non-Western countries. No significant effect size differences were found. Similar subgroup results were found in the analyses that excluded extreme outliers (online Supplementary Table S4).

**Table 4.** Subgroup analyses of psychotherapy for adult depression in Western and non-Western countries

		*N*	*g*	95% CI	*I*^2^	95% CI	NNT	*p*
Category	Non-Western	117	1.10	0.90–1.31	91.1	89.8–92.2	2.49	**<0.001**
	Western	353	0.57	0.52–0.62	75.0	72.3–77.4	5.18	
Geographic region	Asia	92	1.08	0.89–1.26	90.3	88.8–91.7	2.54	**<0.001**
	Latin America	9	0.64	0.31–0.96	77.0	56.1–87.9	4.54	
	Africa	16	1.82	0.39–3.25	94.5	92.5–96.0	1.57	
	North America	134	0.57	0.49–0.65	70.2	64.5–74.9	5.18	
	Europe	183	0.54	0.47–0.62	77.2	73.8–80.1	5.51	
	Oceania	36	0.71	0.52–0.90	73.9	63.8–81.1	4.04	
Word Bank region	East Asia and Pacific	86	0.83	0.67–0.99	86.9	84.4–89.0	3.39	**<0.001**
	Europe and Central Asia	187	0.55	0.48–0.63	77.5	74.2–80.3	5.40	
	Middle East and North Africa	26	1.51	1.14–1.87	86.8	81.8–90.4	1.82	
	South Asia	12	0.85	0.45–1.25	94.5	92.1–96.2	3.30	
	Latin America and Caribbean	9	0.64	0.31–0.96	77.0	56.1–87.9	4.54	
	Sub-Saharan Africa	16	1.82	0.39–3.25	94.5	92.5–96.0	1.57	
	North America	134	0.57	0.49–0.65	70.2	64.5–74.9	5.18	
Income level of country	High	377	0.59	0.53–0.64	76.2	73.8–78.4	4.98	**<0.001**
	Upper middle	50	1.00	0.78–1.21	89.6	87.2–91.6	2.76	
	Low/lower middle	43	1.45	0.93–1.97	93.3	91.8–94.5	1.89	
High income country	High, Western	350	0.57	0.52–0.62	75.1	72.4–77.5	5.18	0.122
	High, non-Western	27	0.81	0.50–1.11	84.9	79.1–89.1	3.48	

*N*, number of comparisons; CI, confidence interval; NNT, numbers-needed-to-be-treated.

#### Multivariate meta-regression

The results of multivariate meta-regression are presented in Table 5. In model 1, whether studies were conducted in Western or non-Western countries remained a significant predictor for the effect size, after adjusting multiple study characteristics (*p* < 0.001). Several predictors were significantly associated with the effect sizes, as a larger effect size was related to the presence of WL, high risk of bias, CBT, and clinician diagnoses (i.e. major depressive disorders, mood disorders, or chronic depression). In model 2, the region of countries was also significantly associated with the effect sizes, along with other predictors (control group, risk of bias, diagnostic methods, and type of therapy). Lastly, the income level remained a significant predictor of the effect sizes, with other predictors being the same as those found in the first model.

**Table 5.** Full multivariate meta-regression analyses of study characteristics on psychotherapy for adult depression in Western and non-Western countries

		Coeff	s.e.	*p*	Coeff	s.e.	*p*	Coeff	s.e.	*p*
Western *vs.* Non-Western countries		0.45	0.08	**<0.001**						
World Bank region	East Asia and Pacific				Ref					
	Europe and Central Asia				−0.22	0.08	**0.01**			
	Middle East and North Africa				0.71	0.16	**<0.001**			
	South Asia				0.19	0.19	0.32			
	Latin America and Caribbean				−0.12	0.21	0.57			
	Sub-Saharan Africa				0.69	0.23	**0.00**			
	North America				−0.27	0.09	**0.00**			
	Income level of country	High							Ref	
	Upper middle							0.29	0.10	**0.005**
	Low/lower middle							0.78	0.12	**<0.001**
Control group	CAU	Ref			Ref			Ref		
	WL	0.27	0.08	**<0.001**	0.24	0.08	**0.00**	0.28	0.08	**<0.001**
	Other	0.05	0.09	0.56	−0.02	0.08	0.81	0.00	0.09	0.99
Risk of bias (continuous)		−0.11	0.03	**<0.001**	−0.13	0.03	**<0.001**	−0.12	0.03	**<0.001**
Age category	Adults	Ref			Ref			Ref		
	Younger adults	0.16	0.18	0.36	0.25	0.18	0.16	0.22	0.18	0.22
	Older adults	−0.06	0.13	0.66	−0.09	0.16	0.50	−0.11	0.16	0.43
Mean age (continuous)		0.00	0.00	0.43	0.01	0.00	0.13	0.01	0.00	0.14
Proportion of women (continuous)		0.21	0.16	0.19	0.24	0.16	0.12	0.20	0.16	0.22
Target group	Unselected adults	Ref			Ref			Ref		
	Women with PPD	−0.11	0.13	0.38	−0.10	0.13	0.44	−0.08	0.13	0.51
	General medical disease	0.01	0.10	0.96	−0.04	0.11	0.73	−0.03	0.10	0.74
	Other	0.08	0.10	0.41	0.05	0.10	0.62	0.06	0.10	0.54
Diagnosis	Cut-off	Ref			Ref			Ref		
	Depressive disorder	0.15	0.07	**0.02**	0.13	0.06	**0.04**	0.15	0.06	**0.02**
	Subclinical	0.12	0.13	0.35	0.17	0.12	0.17	0.19	0.12	0.12
Recruitment	Clinical	Ref			Ref			Ref		
	Community	0.10	0.09	0.27	0.09	0.09	0.30	0.08	0.09	0.38
	Other	0.17	0.10	0.10	0.13	0.10	0.21	0.15	0.10	0.13
Type of treatment	CBT	Ref			Ref			Ref		
	Other	−0.15	0.06	**0.01**	−0.16	0.06	**0.01**	−0.16	0.06	**0.01**
Format of treatment	Group	Ref			Ref			Ref		
	Individual	−0.07	0.08	0.41	−0.05	0.08	0.55	−0.09	0.08	0.23
	Guided self-help	−0.12	0.09	0.20	−0.10	0.09	0.27	−0.15	0.09	0.09
	Other	−0.06	0.11	0.56	0.01	0.11	0.88	−0.06	0.10	0.54
Number of sessions (continuous)		0.04	0.03	0.30	0.05	0.03	0.08	0.04	0.03	0.18
intercept		0.90	0.24	**<0.001**	0.60	0.25	**0.02**	0.43	0.25	0.08
*R*^2^ analog		0.26			0.31			0.28		

Coeff, regression coefficient; s.e., standard error; Ref, reference group; PPD, post-partum depression; CBT, cognitive behavior therapy; CAU, care as usual; WL, wait-list.

To test whether the extreme outliers would affect the results, we repeated the three models and excluded studies with extremely high effect sizes (*g* > 2; online Supplementary Table S5). Our results showed that, in the three models, the effect sizes were additionally associated with the target group, recruitment methods, and number of treatment sessions, with other predictors remaining as in the previous analyses. Finally, when performing the parsimonious multivariate meta-regression, we found that in all three models, the risk of bias and type of therapy remained significant, as well as the variables indicating Western *vs.* no-Western, the regions, and the income levels (online Supplementary Tables S6 and S7).

## Discussion

This study aimed to update a previously published meta-analysis and expand it by identifying potential contributing factors to the larger effect sizes found in non-Western trials. Our findings confirmed previous research, demonstrating that non-Western trials showed significantly larger treatment effects compared to Western trials. This difference even remained after accounting for multiple study characteristics. Furthermore, we identified several notable differences in study characteristics between Western and non-Western trials, including characteristics of trials (type of control group and risk of bias), participants (sample size, mean age, age category, and recruitment method), and treatment (format). Through meta-regression analyses, we shed light on the underlying factors associated with the larger effect sizes observed in non-Western trials. Specifically, our results highlighted the influence of WL control groups, higher risk of bias, implementation of CBT, and depression diagnoses conducted by clinicians.

For the differences in study characteristics, studies conducted in non-Western countries more frequently utilized CAU as a control group. They also included a higher proportion of LMICs and had a higher risk of bias. These findings were consistent with previous research (Cuijpers et al., 2018). Additionally, we found that the elevated risk of bias observed in non-Western trials was primarily attributed to improper analyses involving missing data, with less emphasis on ITT approaches. In terms of participant recruitment, non-Western countries relied more on alternative methods rather than communities or clinical settings. This outcome may reflect the limited availability of mental health care centers in non-Western countries, as evidence showed that Asia had fewer mental health facilities than Europe and North America (Ito, Setoya, & Suzuki, 2012). Moreover, psychotherapies implemented in non-Western trials were more frequently adopted in a group-based format, while individual-based interventions were predominant in Western trials. This result aligns with our observations that non-Western trials had larger sample sizes, which may be more conducive to group-based interventions. It may also indicate the scarcity of psychiatrists (1.55 per 10 000) available for individual-based treatment in LMICs compared to HICs (3.96 per 10 000; Cuijpers, 2022; Rathod et al., 2017).

For the factors contributing to the higher effect sizes observed in non-Western studies, our findings were consistent with previous research, showing that the larger effects in non-Western countries remained significant after adjusting for the type of control group (Cuijpers et al., 2018). Moreover, we found that the high risk of bias, which presented more in non-Western trials, could result in a larger effect size (Cuijpers et al., 2018). When comparing clinician-assessed depressive disorders to self-rating questionnaires, we found that the former had larger effect sizes, indicating that self-report measures are either more conservative or less sensitive to change. It may also be a combination of both (Cuijpers et al., 2010). Lastly, our results aligned with previous findings indicating that CBT yielded better outcomes compared to alternative therapies (Tolin, 2010); however, it contrasted studies that showed comparable effects across different types of psychotherapy (Cuijpers et al., 2017, 2021b).

Regarding the different results found in analyses with *vs.* without extreme outliers, the results that excluded extreme outliers suggested a potential association between the age category and larger effect size. These results align with previous findings, showing that college students had relatively high treatment effects (*g* = 0.89) than unselected adults (*g* = 0.79; Cuijpers et al., 2016), although the differences were not statistically significant. More importantly, the parsimonious meta-regression analyses found the same predictors in both analyses with and without extreme outliers. These findings indicated that the validity of trials and the type of psychotherapies appeared to be the most influential factors contributing to the effect sizes.

### Limitations and strengths

Several limitations of the current paper were notable, and further studies are needed. First, this study only measured the effect sizes at post-assessments and only for adults, while it remains unknown whether the larger effect observed in non-Western trials could last in the long term and how it applies to children and adolescents. In addition, this study did not assess the effects of individual-level factors, such as the baseline severity of depression. It is possible that current findings would be moderated by the baseline depressive severity, especially given the high heterogeneity of included participants. Lastly, we were unable to explain the different findings in the analyses with *vs.* without extreme outliers. The full analyses that included extreme outliers may reflect the true differences in study characteristics, or they may imply the methodological issues raised by studies with effect sizes that were significantly higher than expected.

Nevertheless, this meta-analysis identified twice as many RCTs as the previous paper, thereby increasing the statistical power to confirm former findings (Cuijpers et al., 2018). It is also the first study that compared different study characteristics in Western and non-Western trials, and revealed how these characteristics can affect treatment effect estimates. Our results showed significant heterogeneity in study characteristics across non-Western trials, as well as a disparity in the risk of bias between Western and non-Western countries. Therefore, the larger treatment effects observed in non-Western trials may not necessarily imply superior treatment outcomes. On the other hand, it could stem from variations in study design and quality. These findings also underscore the significant mental health gap between Western and non-Western countries, carrying important implications not just for the researchers but for the clinicians and policymakers (Patel, 2007; WHO, 2022). Specifically, it may be advisable for clinicians in non-Western countries to prioritize the delivery of CBT over other types of psychotherapy. Additionally, the higher risk of bias identified in non-Western trials suggests the need for increased attention to ensure high-quality research in these regions. Policymakers could consider providing training and support for researchers in non-Western countries to enhance the validity and rigor of their research methods.

## Conclusion

This meta-analysis confirmed previous results stating that studies from non-Western countries had a larger effect size than those from Western countries. Moreover, our findings are novel in showing differences in study characteristics between Western and non-Western trials and, more importantly, how these differences can result in a larger treatment effect size. In light of our findings, the perceived larger treatment effects for adult depression observed in non-Western countries may be due to differing study characteristics and validity. Further research is required to explain the reasons for the differences in study design and quality between Western and non-Western trials, as well as the different results in the analyses with and without extreme outliers. Research focusing on long-term effects, children and adolescents, and individual-level factors are also required.

## Supporting information

Tong et al. supplementary material 1 Tong et al. supplementary material

Tong et al. supplementary material 2 Tong et al. supplementary material

## Supplementary material

The supplementary material for this article can be found at https://doi.org/10.1017/S0033291723002246.
