# Supplementary material for: Psychotherapy for adult depression in low- and middle-income countries: an updated systematic review and meta-analysis
Source: Psychol Med. 2023 Aug 23;53(16):7473–83. doi: 10.1017/S0033291723002246 (PMC10951412; doi:10.1017/S0033291723002246)
Supplement: Tong et al. supplementary material 2 — Tong et al. supplementary material [file S0033291723002246sup002.html]

Maps of studies per country


# Maps of studies per country

#### 

#### 2023-06-13
